# Circulating Thioredoxin 1 as an Adjunct to Mammography for Breast Cancer Detection: A Multicenter Clinical Validation Study

**DOI:** 10.3390/cancers18152416

**Published:** 2026-07-27

**Authors:** Hye Mi Ko, Jee Ye Kim, Songhak Kim, Jungchan Shin, Xiaoguang Yang, Jong Am Song, Ji Yeon Kim, Sang Il Lee, Jeong Eun Lee, Bo Bae Choi, Jin Man Kim, Jin Gyu Jung, Je Ryong Kim, Ji Young Sul, Eun Heui Jin, Jang Hee Hong, Choong Sik Lee, Kyoung Hoon Suh, Seung Il Kim, Jin Sun Lee

**Affiliations:** 1 Department of Surgery, Chungnam National University Hospital & Chungnam National University School of Medicine, Daejeon 35015, Republic of Korea; camomill@cnuh.co.kr (H.M.K.); jkim@cnu.ac.kr (J.Y.K.); mr231@cnuh.co.kr (S.I.L.); kimjr@cnuh.co.kr (J.R.K.); jysul@cnu.ac.kr (J.Y.S.); 2 Department of Surgery, Severance Hospital, Yonsei University College of Medicine, Seoul 03722, Republic of Korea; jeeye0531@yuhs.ac (J.Y.K.); seungil@yuhs.ac (S.I.K.); 3 E&S Healthcare, Daejeon 34002, Republic of Korea; shkim85@ens-h.com (S.K.); jcshin@ens-h.com (J.S.); yangxg@ens-h.com (X.Y.); sja@ens-h.com (J.A.S.); one@ens-h.com (K.H.S.); 4 Department of Internal Medicine, College of Medicine, Chungnam National University, Daejeon 35015, Republic of Korea; jelee0210@cnu.ac.kr; 5 Department of Radiology, Chungnam National University Hospital, Daejeon 35015, Republic of Korea; med20@cnuh.co.kr; 6 Department of Pathology, Chungnam National University Hospital, Daejeon 35015, Republic of Korea; jinmank@cnuh.co.kr; 7 Department of Family Medicine, Chungnam National University Hospital, Daejeon 35015, Republic of Korea; jjg72@cnuh.co.kr; 8 Department of Pharmacology, Chungnam National University College of Medicine, Daejeon 35015, Republic of Korea; sowha1219@cnuh.co.kr (E.H.J.); boniii@cnu.ac.kr (J.H.H.); 9 Department of Pathology, Konyang University Hospital, Daejeon 35365, Republic of Korea; cslee@kyuh.ac.kr

**Keywords:** thioredoxin 1, breast cancer, circulating biomarker, dense breast, mammography, decision curve analysis

## Abstract

**Background/Objectives**: Mammography remains central to breast cancer diagnosis, yet clinically meaningful uncertainty persists in women with dense breasts and in patients with very small lesions or indeterminate imaging findings. We evaluated whether circulating thioredoxin 1 (Trx1) could provide biological information that complements imaging-based assessment. **Methods**: This multicenter clinical validation study evaluated circulating Trx1 in 1901 serum samples across four predefined cohorts. Diagnostic performance was assessed using receiver operating characteristic (ROC) and precision–recall analyses, together with evaluation of integration with mammographic assessment and decision curve analysis (DCA). A predefined Trx1 cutoff established in prior clinical investigations was applied. **Results**: Circulating Trx1 concentrations were significantly elevated in breast cancer compared with controls (*p* < 0.001). Trx1 showed excellent diagnostic discrimination (AUC 0.985; 95% CI, 0.974–0.996), with sensitivity and specificity of 96.4% and 97.3%, respectively. Diagnostic performance was consistent across disease stages, tumor-size categories, molecular subtypes, mammographic categories, and breast-density groups. Trx1 sensitivity remained high in dense breasts (98.9% in BI-RADS density grades C/D), whereas mammographic sensitivity was substantially lower (67.0%). Integration of Trx1 with mammographic assessment improved diagnostic discrimination (AUC 0.980) and provided greater net benefit in decision curve analysis, particularly among women with low-suspicion or indeterminate imaging findings. **Conclusions**: Circulating Trx1 demonstrated robust diagnostic performance across disease stages, tumor-size categories, and clinically relevant patient subgroups while providing biologically independent information that complements mammographic assessment. Although this retrospective multicenter study requires prospective validation in independent diagnostic populations, Trx1 may serve as a clinically useful adjunct to imaging-based breast cancer evaluation, particularly in diagnostically uncertain settings.

## Simple Summary

Breast cancer is commonly detected using mammography, but the accuracy of imaging can be reduced in women with dense breast tissue and in patients with very small tumors or inconclusive imaging findings. Blood-based biomarkers may provide additional information and improve diagnostic assessment in these situations. In this study, we evaluated thioredoxin 1, a protein associated with breast cancer biology, as a complementary blood biomarker in a multicenter cohort of 1901 serum samples. We found that circulating thioredoxin 1 distinguished breast cancer from non-cancer controls and showed strong diagnostic performance across different tumor stages, tumor sizes, molecular subtypes, and breast-density categories. When combined with mammographic assessment, thioredoxin 1 improved diagnostic performance and demonstrated greater net benefit in decision curve analysis, particularly in women with dense breasts and in patients with indeterminate imaging findings. These findings suggest that thioredoxin 1 may serve as a clinically useful adjunct to imaging-based breast cancer evaluation.

## 1. Introduction

Mammography remains the cornerstone of breast cancer (BC) screening and has contributed substantially to reductions in BC mortality [1]. Advances in imaging technologies have improved diagnostic performance; however, a major limitation persists in women with dense breast tissue [2]. In these populations, the masking effect, in which tumors and fibroglandular tissue share similar radiographic appearances, substantially reduces mammographic sensitivity, with reported values falling below 60% in some settings [3,4,5]. Interval cancers occur more frequently in women with dense breasts, and breast density itself is an established independent risk factor for BC development [6,7,8,9]. In addition, very small lesions, non-calcified tumors, and cancers presenting with low-suspicion or indeterminate imaging findings may be more difficult to detect reliably using imaging alone [10,11]. As biomarker concentrations frequently increase with tumor growth and disease progression, diagnostic performance may be reduced in early-stage disease and in patients with small tumors, where circulating biomarker levels are often less pronounced [12,13]. Together, these factors create the dual challenge of increased disease risk and reduced detectability, suggesting that improvements in imaging alone may be insufficient to fully resolve diagnostic uncertainty.

Serum tumor markers have long been explored as potential tools for BC detection. Currently available circulating biomarkers, including CA15-3 and CEA, have limited utility for primary BC diagnosis because of insufficient sensitivity and specificity, particularly in early-stage disease [13,14,15]. Consequently, neither marker is recommended for screening or diagnosis. These limitations have prompted continued investigation into alternative classes of circulating biomarkers.

Oxidative stress is increasingly recognized as an important contributor to cancer development, progression, and therapeutic resistance [16]. Elevated levels of reactive oxygen species (ROS) promote genomic instability, metabolic reprogramming, and aberrant cell signaling, while cancer cells simultaneously activate antioxidant defense systems to maintain redox homeostasis [17,18]. Among these antioxidant defense systems, the thioredoxin system is a major regulator of cellular redox balance and has been implicated in multiple aspects of tumor biology, including cell proliferation, apoptosis, angiogenesis, and resistance to therapy [19,20,21,22,23]. Owing to its central role in maintaining redox homeostasis and its frequent dysregulation in cancer, the thioredoxin system has attracted considerable interest as a source of blood-based cancer biomarkers.

Thioredoxin 1 (Trx1), the major active component of the thioredoxin system, is a redox-active protein involved in oxidative stress regulation and has been implicated in multiple malignancies [19,20,21,22,23]. In breast cancer, previous studies reported Trx1 overexpression at the tissue level [24] and subsequently reported elevated circulating concentrations [25], supporting its potential as a blood-based biomarker for BC detection [26]. Progressive refinement of the assay platform across successive studies has further improved its diagnostic performance.

We therefore hypothesized that Trx1 could function as a blood-based adjunct to mammography and improve diagnostic performance in clinically challenging contexts where imaging sensitivity is limited. In this multicenter study, we evaluated whether integration of circulating Trx1 with mammographic assessment enhances BC detection, with particular emphasis on women with dense breasts.

## 2. Materials and Methods

### 2.1. Study Design and Participant Recruitment

This multicenter clinical study included participants recruited from tertiary hospitals and commercial biobanks to evaluate circulating Trx1 in breast cancer detection. The clinical cohort comprised women enrolled during diagnostic evaluation for breast disease, including patients with histologically confirmed breast cancer, benign breast disease, and healthy controls. Eligible archived serum specimens stored in institutional biobanks for up to three years were randomly selected after application of the predefined inclusion and exclusion criteria. Serum samples from clinically enrolled participants were collected after histopathological confirmation of diagnosis and before initiation of any cancer-directed treatment. These samples were used for the primary diagnostic performance and imaging-integrated analyses.

Additional de-identified serum samples were obtained from commercial biobanks, including Asterand Biosciences (Detroit, MI, USA), Folio Conversant Bio (Huntsville, AL, USA), and Discovery Life Sciences (Huntsville, AL, USA). Biobank-derived samples included breast cancer, other malignancies, non-cancer diseases, and healthy controls and were included exclusively to evaluate the intrinsic diagnostic performance of circulating Trx1 across a broad spectrum of malignant and non-malignant conditions, independent of imaging findings. Following receipt, all commercial specimens were immediately aliquoted, stored at −70 °C, and analyzed within the documented storage validity period specified by the supplier. All Trx1 measurements were performed using the same analytical assay in a single central laboratory to minimize freeze–thaw cycles and analytical batch variation. The final analytical dataset comprised 1901 serum samples.

### 2.2. Analytical Cohort Definitions

To ensure transparency in participant inclusion across analyses, four predefined analytical cohorts were established before data analysis according to the study objectives.

(I) Primary diagnostic performance cohort (*n* = 644)

The primary diagnostic performance cohort comprised 644 clinically enrolled participants, including 308 patients with histologically confirmed breast cancer, 82 patients with benign breast disease, and 254 healthy controls. This cohort was used to evaluate the intrinsic diagnostic performance of circulating Trx1 independent of imaging findings, including receiver operating characteristic (ROC) analysis, sensitivity, specificity, positive predictive value (PPV), negative predictive value (NPV), precision–recall analysis, and stage-stratified performance assessment.

(II) Density-stratified performance cohort (*n* = 261 and 258)

The density-stratified performance cohort comprised 261 participants with available breast-density information obtained from ultrasonographic assessment. This cohort was used to evaluate Trx1 sensitivity across breast-density categories and to compare diagnostic performance between Trx1, conventional biomarkers, and an integrated mammography–Trx1 approach according to breast density. Comparisons with CA15-3 and CEA were performed in 258 participants after exclusion of 3 individuals lacking measurements.

(III) Imaging comparison cohort (*n* = 582 and 597)

For ROC-based comparison of mammography and Trx1, participants classified as BI-RADS 0 or BI-RADS 6 were excluded because these categories represent incomplete imaging assessment and biopsy-proven malignancy, respectively. The resulting cohort included 582 participants, comprising 254 patients with breast cancer and 328 non-breast cancer controls.

For decision curve analysis (DCA), BI-RADS 6 cases were excluded because these lesions had already been histopathologically confirmed as breast cancer and therefore do not represent clinically relevant diagnostic decision-making scenarios. In contrast, BI-RADS 0 cases were retained to reflect clinically indeterminate imaging scenarios, where an adjunctive biomarker may provide additional diagnostic information. This analysis included 597 participants, comprising 261 patients with breast cancer and 336 non-breast cancer controls.

(IV) Cross-disease specificity cohort (*n* = 1901)

The cross-disease specificity cohort comprised the full study population, including all clinically enrolled participants and biobank-derived samples. This cohort was used to characterize circulating Trx1 concentrations across breast cancer, other malignancies, non-cancer diseases, benign breast conditions, and healthy controls, and to evaluate disease specificity beyond breast cancer detection.

### 2.3. Imaging-Based Clinical Evaluation

Imaging followed routine clinical practice at each site. Mammographic findings were categorized according to the Breast Imaging Reporting and Data System (BI-RADS), and breast density was graded by a board-certified radiologist as A (fatty), B (scattered), C (heterogeneously dense), or D (extremely dense). BI-RADS 0–3 categories were defined as representing diagnostically uncertain or low-suspicion findings, whereas BI-RADS 4–5 were considered indicative of higher suspicion for malignancy. For the combined mammography–Trx1 approach, participants were classified as positive when either mammographic assessment or circulating Trx1 yielded a positive result.

### 2.4. Protein Quantification

Circulating Trx1 was measured using the DxMe^®^ BC ELISA kit (E&S Healthcare, Republic of Korea), following our previously published protocol [26]. The diagnostic cut-off value for Trx1 was predefined based on a prior exploratory clinical study and was applied unchanged in the present analysis. To evaluate the independent diagnostic contribution of circulating Trx1, multivariable logistic regression analysis was performed with breast cancer status as the dependent variable and age, BI-RADS category, and circulating Trx1 concentration as independent variables. CA15-3 (Elecsys CA15-3 II, Roche, Indianapolis, IN, USA) and CEA (Elecsys CEA, Roche, Indianapolis, IN, USA) were measured by ECLIA on the Cobas e801 analyzer (Roche, Indianapolis, IN, USA) following the manufacturers’ protocols. All serum samples from participating centers were processed and analyzed in a single central laboratory; laboratory personnel were blinded to clinical outcomes throughout.

### 2.5. Decision Curve Analysis

Potential clinical utility was assessed using DCA. Net benefit was defined as:

$$Net\;Benefit=\frac{TP}{N}-\frac{FP}{N}\times \frac{p_{t}}{1-p_{t}}$$

where TP and FP represent true-positive and false-positive classifications, N is the total number of individuals, and pt denotes the selected threshold probability. Net benefit was calculated across a range of threshold probabilities from 0.01 to 0.30, following the framework described by Vickers [27,28]. This range was selected to reflect clinically relevant probability thresholds at which additional diagnostic evaluation would typically be considered in breast cancer assessment. Net reduction in unnecessary diagnostic interventions per 100 patients was derived from the same framework across the same threshold range. Net reduction was calculated across the same threshold probability range (0.01–0.30) and derived from the net benefit estimates obtained from the DCA. The threshold probability represents the risk level at which additional diagnostic evaluation would be recommended.

### 2.6. Statistical Analysis

ROC curve analysis was performed using MedCalc (version 19.1.5; MedCalc Software, Ostend, Belgium) to determine the sensitivity, specificity, and area under the curve (AUC) of Trx1 for distinguishing breast cancer from non-breast cancer controls. Group comparisons were conducted using the Kruskal–Wallis test, one-way ANOVA, or unpaired *t*-tests, as appropriate. A two-sided *p* value < 0.05 was considered statistically significant. Missing data were not imputed. Analyses were performed using complete-case datasets, and participants with missing variables required for a specific analysis were excluded from that analysis. The sample size for the primary diagnostic performance cohort was determined before study initiation based on published mammographic sensitivity and specificity. In contrast, the disease-specificity cohort was determined according to the availability of archived biobank specimens.

## 3. Results

### 3.1. Study Cohorts and Baseline Clinicopathologic Characteristics

**Figure 1:**
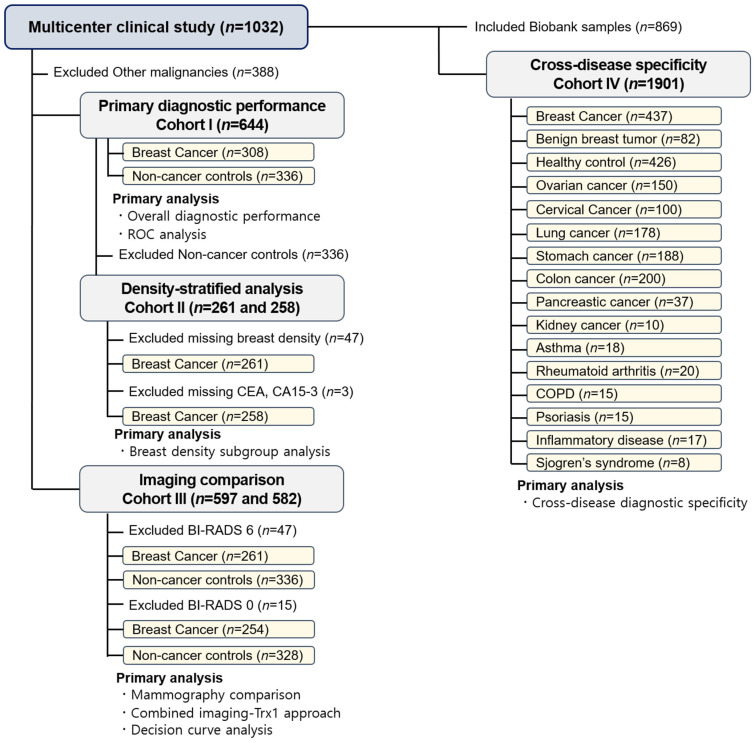
Study design and analytical cohort definitions. Flow diagram illustrating participant enrollment and allocation into four predefined analytical cohorts. Cohort I was used to evaluate the overall diagnostic performance of circulating Trx1 for breast cancer detection. Cohort II included participants with available breast-density information and was used for density-stratified analyses. Cohort III was used to compare the diagnostic performance of Trx1, mammography, and the combined mammography–Trx1 approach. Cohort IV comprised patients with breast cancer, other malignancies, and non-malignant conditions and was used to assess cross-disease specificity. The number of participants included in each cohort and reasons for exclusion are shown.

This study consisted of four analytical cohorts, as summarized in Figure 1. The primary diagnostic cohort included 308 patients with BC, 82 patients with benign breast tumor, and 254 healthy controls, collectively comprising the non-breast cancer (NBC) group, and was used for the primary evaluation of the diagnostic performance of Trx1 in BC. The baseline demographic characteristics of this cohort are summarized in Supplementary Table S1.

Among women with normal findings or benign breast tumors, circulating Trx1 concentrations did not differ significantly according to age group (*p* = 0.633) and showed no meaningful variation across mammographic BI-RADS categories (*p* = 0.364), and remained relatively stable throughout BI-RADS categories 0–3. In contrast, BC patients exhibited markedly elevated circulating Trx1 concentrations compared with non-cancer groups across all examined subcategories (Table 1).

Within the breast cancer cohort, circulating Trx1 concentrations were not significantly associated with age (*p* = 0.265) or mammographic BI-RADS category (*p* = 0.155). Similarly, stratification according to breast density showed comparable circulating Trx1 concentrations across all breast-density grades, with no significant intergroup differences (*p* = 0.355) (Table 1).

### 3.2. Intrinsic Diagnostic Performance of Circulating Trx1

**Figure 2:**
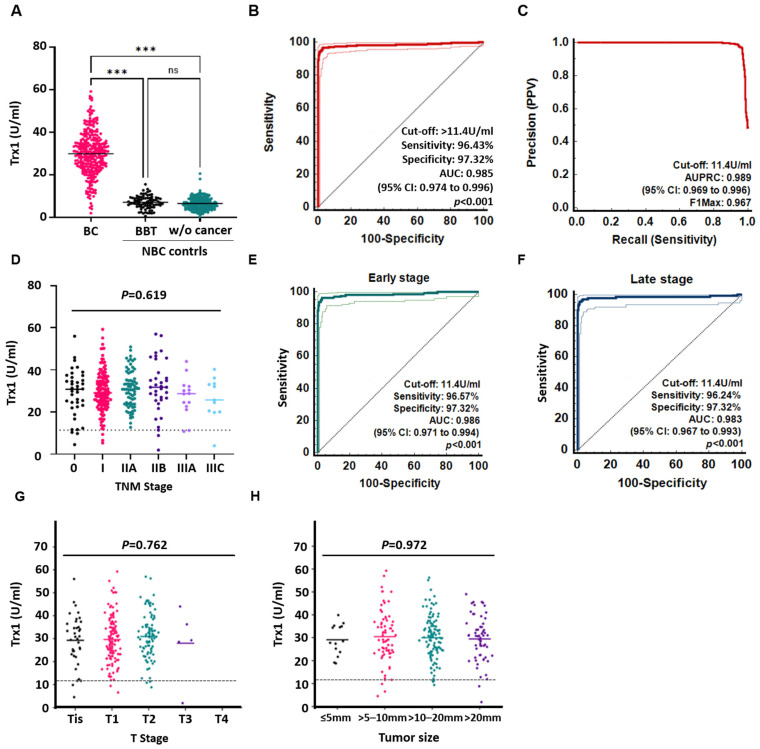
Diagnostic performance of circulating Trx1 in breast cancer. (**A**) Distribution of circulating Trx1 concentrations in breast cancer and non-cancer controls. (**B**) Receiver operating characteristic (ROC) analysis demonstrating the diagnostic performance of Trx1 for discrimination of breast cancer from controls. (**C**) Precision–recall analysis evaluating classification performance in the study population. (**D**) Circulating Trx1 concentrations according to TNM stage. (**E**,**F**) Stage-specific ROC analyses for early-stage (stage 0–I) and late-stage (stage II–III) breast cancer, respectively. (**G**) Distribution of circulating Trx1 concentrations according to pathological T category. (**H**) Distribution of circulating Trx1 concentrations according to tumor-size category. The dashed horizontal line indicates the predefined diagnostic cutoff value of 11.4 U/mL. AUPRC, area under the precision–recall curve. In panels B, C, E, and, F, the solid curves represent the estimated ROC or precision-recall curves, and the lighter curves indicate the corresponding 95% confidence intervals. Statistical significance is indicated as *** *p* < 0.001; ns, not significant.

The intrinsic diagnostic performance of circulating Trx1 was assessed in the primary diagnostic performance cohort (I), independently of mammographic findings. Accordingly, all eligible BC cases, including BI-RADS 0 and 6, were included to assess the overall diagnostic performance of circulating Trx1 irrespective of imaging findings. Analyses included sensitivity, specificity, and ROC performance, and the results are presented in Figure 2, Table 2, and Supplementary Table S2. Circulating Trx1 concentrations were significantly higher in BC patients than in NBC controls (*p* < 0.001) (Figure 2A).

At the predefined cut-off of 11.4 U/mL, Trx1 showed sensitivity of 96.43% (95% CI: 93.7–98.2%) and specificity of 97.32% (95% CI: 95.0–98.8%), with an AUC of 0.985 (95% CI: 0.974–0.996) (Figure 2B). Precision–recall analysis confirmed robust predictive performance across the full range of classification thresholds: AUPRC 0.989 (95% CI: 0.969–0.996), PPV 97.06% (95% CI: 94.5–98.4%), and NPV 96.75% (95% CI: 94.3–98.2%) (Figure 2C).

Circulating Trx1 concentrations remained elevated across tumor size, TNM stage, histologic grade, molecular subtype, and other biological subgroups, with no significant intergroup differences (Table 2). Likewise, Trx1 concentrations showed no significant variation across disease stages and remained elevated from Stage 0 through Stage III disease (Figure 2D). Stage-specific ROC analyses showed similarly high diagnostic accuracy for early-stage (0–I) and more advanced-stage (II–III) BC, with AUC values of 0.986 and 0.983, respectively (Figure 2E,F). Detailed performance metrics are summarized in Table 2.

Additional analyses were performed to evaluate whether circulating Trx1 concentrations were influenced by pathological tumor category or tumor size. Trx1 concentrations remained elevated across pathological T categories, including Tis, T1, T2, and T3 tumors, without significant intergroup differences (*p* = 0.762) (Figure 2G). Similarly, Trx1 concentrations showed no significant variation across tumor-size categories (<5 mm, 5–10 mm, 10–20 mm, and >20 mm; *p* = 0.972) and remained substantially above the predefined diagnostic cutoff even in tumors measuring <5 mm (Figure 2H). Performance metrics according to pathological T category and tumor size are provided in Table 2.

### 3.3. Circulating Trx1 as a Complementary Biomarker for Imaging-Based Breast Cancer Diagnosis

**Figure 3:**
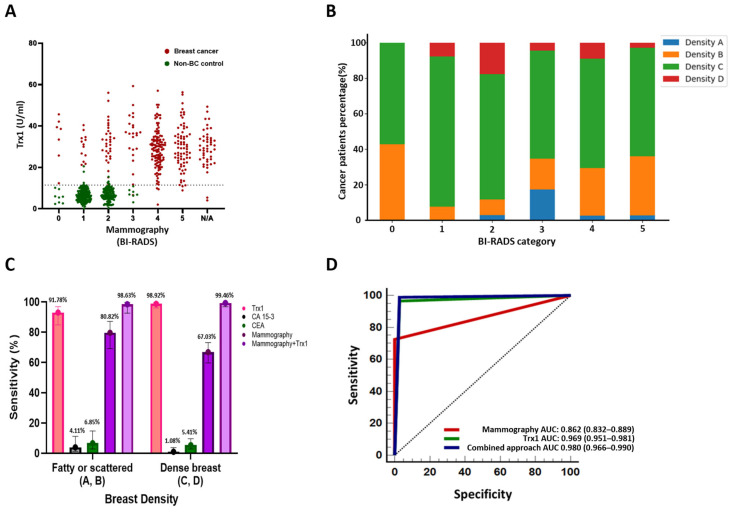
Performance of circulating Trx1 according to mammographic assessment and breast density. (**A**) Distribution of circulating Trx1 concentrations across BI-RADS categories. The dashed horizontal line indicates the predefined diagnostic cutoff value of 11.4 U/mL. (**B**) Distribution of BI-RADS categories according to breast density, illustrating the increased frequency of low-suspicion and indeterminate imaging findings among women with dense breasts. (**C**) Sensitivity of Trx1, CA15–3, CEA, mammography, and the combined mammography–Trx1 approach across breast-density categories. Trx1 maintained high sensitivity across density groups, whereas mammographic sensitivity declined with increasing breast density. (**D**) ROC analysis comparing mammography alone, Trx1 alone, and the combined mammography–Trx1 approach. Integration of Trx1 with mammographic assessment improved diagnostic discrimination relative to mammography alone. BI-RADS, Breast Imaging Reporting and Data System.

Given the stable diagnostic performance of Trx1 across clinicopathological subgroups, we next examined its behavior in imaging contexts where mammographic assessment is known to be limited. This analysis was designed to compare the diagnostic performance of mammographic assessment, circulating Trx1, and the combined mammography–Trx1 approach. Therefore, BI-RADS 6 cases, which had already been histopathologically confirmed as BC, were excluded from the imaging comparison cohort. To this end, we evaluated the distribution of initial BI-RADS categories within the imaging comparison cohort. All NBC participants were classified within BI-RADS categories 0–3. Notably, among the 308 BC patients, 77 were also initially assigned BI-RADS categories 0–3 on mammography. Circulating Trx1 concentrations remained elevated in BC patients across all BI-RADS categories, including those associated with indeterminate or low-suspicion mammographic findings, whereas participants without BC had substantially lower concentrations throughout these categories (Figure 3A).

Within the density-stratified cohort (II), we further evaluated the relationship between breast density and initial BI-RADS classification (Figure 3B). Among the 261 subjects included in this analysis, Grade C breast density constituted the largest proportion (*n* = 166, 63.6%). Among cases classified as BI-RADS 0–3, most participants had higher breast density (Grades C/D), whereas low-density breasts (Grades A/B) were comparatively uncommon. The distribution of BI-RADS categories according to breast density is summarized in Supplementary Table S3. Across breast-density Grades A–D, we compared the diagnostic performance of Trx1, CA15-3, CEA, mammography, and combined mammography–Trx1 approach (Figure 3C). Trx1 showed high sensitivity across density categories, reaching 91.78% in Grades A/B and 98.92% in Grades C/D. Mean Trx1 concentrations remained relatively stable across density groups (28.50 ± 9.95 to 33.83 ± 13.36 U/mL). Mammographic sensitivity, defined as the proportion of BC cases initially classified as BI-RADS 4–5, varied substantially according to breast density, ranging from 50.00% in Grade A and 85.71% in Grade B to 67.88% in Grade C and 60.00% in Grade D (Supplementary Table S4). The combined mammography–Trx1 approach showed the highest sensitivity across all density grades, with the greatest relative improvement observed in dense breasts (Grades C/D).

In the imaging comparison cohort (III; *n* = 582), ROC analysis showed an AUC of 0.862 (95% CI, 0.832–0.889) for mammography alone, 0.969 (95% CI, 0.951–0.981) for circulating Trx1 alone, and 0.980 (95% CI, 0.966–0.990) for the combined mammography–Trx1 approach (Figure 3D and Supplementary Table S5).

To further evaluate the independent diagnostic contribution of circulating Trx1, we performed a multivariable logistic regression analysis including age, BI-RADS category, and circulating Trx1 (Supplementary Table S7). After adjustment for age and BI-RADS category, circulating Trx1 remained independently associated with BC, supporting the independent diagnostic contribution of circulating Trx1 beyond conventional imaging assessment.

### 3.4. Decision Curve Analysis of Diagnostic Strategies

**Figure 4:**
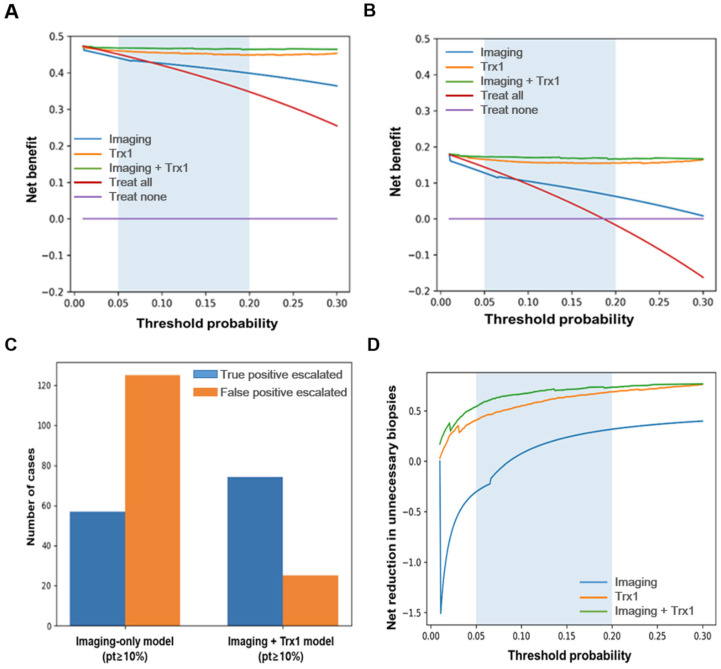
Decision curve analysis of circulating Trx1 within imaging-based breast cancer assessment. (**A**) DCA comparing mammography alone, Trx1 alone, and the combined mammography–Trx1 approach in the imaging comparison cohort (BI-RADS 0–5). (**B**) DCA restricted to women with low-suspicion or indeterminate mammographic findings (BI-RADS 0–3), a subgroup in which diagnostic uncertainty is greater. (**C**) DCA at a threshold probability of 10%, demonstrating the projected numbers of true-positive and false-positive classifications for each diagnostic strategy. (**D**) Net reduction in unnecessary interventions associated with incorporation of Trx1 into imaging-based assessment. Decision curve analysis and net reduction calculations were performed across the threshold probability range of 0.01–0.30. The shaded region indicates the threshold probability range of 0.05–0.20, which was highlighted because it represents the range considered most clinically relevant for decisions regarding additional diagnostic evaluation.

DCA was performed in the imaging comparison cohort (III; *n* = 597) to estimate the potential clinical utility of Trx1 in supporting imaging-based diagnostic decision-making. As in the imaging comparison analysis, BI-RADS 6 cases were excluded because these lesions had already been pathologically confirmed and therefore did not represent clinically relevant diagnostic decision-making scenarios, whereas BI-RADS 0 cases were retained to reflect clinically indeterminate imaging findings. Net benefit was evaluated across threshold probabilities ranging from 0.01 to 0.30 for imaging alone, Trx1 alone, and the combined mammography–Trx1 approach (Figure 4). In the full BI-RADS 0–5 cohort, differences between strategies were relatively modest because imaging-based assessment already showed strong discrimination in higher BI-RADS categories (Figure 4A).

In contrast, within the BI-RADS 0–3 subgroup, which included participants with incomplete, negative, or probably benign imaging findings, strategies incorporating Trx1 provided greater net benefit than imaging assessment alone across much of the evaluated threshold range. (Figure 4B). The combined mammography–Trx1 approach provided the greatest net benefit across most evaluated threshold probabilities. At a threshold probability of 10%, incorporation of Trx1 into imaging assessment increased true-positive detection while reducing false-positive classifications compared with imaging alone (Figure 4C). Strategies incorporating Trx1 were associated with a greater reduction in unnecessary diagnostic interventions across the evaluated threshold range (Figure 4D).

### 3.5. Cross-Disease Specificity of Trx1 Protein

To evaluate the disease specificity of circulating Trx1 across a broad spectrum of diseases, circulating Trx1 concentrations were compared across the cross-disease specificity cohort (IV; *n* = 1901), including all clinically enrolled participants and biobank-derived samples. This analysis included BC, NBC, other malignancies (ovarian, cervical, lung, stomach, colon, pancreatic, and kidney cancers), and inflammatory conditions (Supplementary Figure S2 and Table S6). Breast cancer exhibited the highest circulating Trx1 concentrations among all groups examined (all pairwise comparisons *p* < 0.001, except kidney cancer *p* < 0.01). Although elevated Trx1 concentrations were also observed in several other malignancies, the magnitude of elevation was greatest in BC.

## 4. Discussion

In this multicenter study, circulating Trx1 showed strong diagnostic performance for BC and provided information complementary to mammographic assessment. Three principal findings emerged. First, Trx1 showed high diagnostic accuracy across a broad spectrum of BC patients, including early-stage disease, and performed consistently across disease stages, tumor-size categories, and clinicopathological subgroups (Figure 2). Second, unlike mammography, Trx1 performance was largely unaffected by breast density, with sensitivity remaining high across density categories (Figure 3A,C). Third, integration of Trx1 with mammographic assessment improved overall diagnostic discrimination (Figure 3C,D) and provided greater net benefit in decision curve analysis among patients with low-suspicion or indeterminate imaging findings (Figure 4). Together, these findings suggest that circulating Trx1 provides biological information that complements mammographic assessment and may help address diagnostic challenges in settings where cancer detectability is reduced.

One of the most notable findings was the remarkable consistency of circulating Trx1 concentrations across clinically relevant patient subgroups. From a biomarker validation perspective, preservation of diagnostic performance across heterogeneous patient populations is critical for clinical implementation [29,30]. Trx1 concentrations remained elevated irrespective of disease stage, molecular subtype, Ki-67 expression, histologic grade, or p53 status (Table 2). Similar findings were observed across pathological T categories and tumor-size groups, with no significant intergroup differences in circulating Trx1 concentrations (Figure 2G,H). A major limitation of many circulating cancer biomarkers is their dependence on tumor burden. Biomarker concentrations often increase progressively as tumors enlarge and disease advances, resulting in reduced sensitivity in biologically early cancers [12,13]. In contrast, circulating Trx1 remained positive across disease stages and tumor-size categories in the present study. This pattern is consistent with our previous observations that circulating Trx1 levels are elevated across diverse BC subgroups and are not strongly influenced by clinicopathological characteristics [26]. The reproducibility of this finding in the present multicenter cohort further supports the robustness and generalizability of Trx1 as a diagnostic biomarker across heterogeneous BC populations. Trx1 is a central regulator of cellular redox homeostasis and is induced in response to oxidative stress [31]. This biological role may partly explain the reproducible elevation of circulating Trx1 observed across clinically relevant BC subgroups.

Recent advances in liquid biopsy have expanded the repertoire of blood-based biomarkers for breast cancer detection, including circulating tumor DNA (ctDNA), methylation-based assays, circulating tumor cells (CTCs), extracellular vesicles, and microRNAs [32,33,34]. These emerging biomarkers have shown considerable promise for cancer detection and disease monitoring. However, many rely on technically demanding workflows, specialized instrumentation, and complex bioinformatic analyses, which may limit their widespread implementation in routine clinical practice. Within this evolving diagnostic landscape, circulating Trx1 represents a biologically distinct biomarker that reflects alterations in cellular redox homeostasis rather than tumor-derived genetic or epigenetic changes. Consequently, Trx1 may provide complementary biological information alongside mammographic assessment and other emerging blood-based biomarkers, particularly in diagnostically challenging clinical settings.

Across multiple tumor types, Trx1 participates in adaptive responses to hypoxia, oxidative stress, and metabolic stress, including HIF-1α stabilization, MAPK/ASK1 regulation, and metabolic reprogramming [35,36,37,38]. Recent studies have further highlighted the importance of the Trx1/TXNIP regulatory axis in breast cancer biology. TXNIP, the principal endogenous inhibitor of Trx1, has been implicated in tumor progression, metabolic adaptation, tumor–microenvironment interactions, and endocrine resistance through IGF-1-dependent signaling pathways [39,40]. Dysregulation of this redox regulatory network may contribute not only to BC progression but also to the sustained elevation of circulating Trx1 observed across clinically relevant disease stages. A possible explanation for the preferential elevation of circulating Trx1 in BC lies in the biological context in which these pathways are activated. Breast cancers develop within a dense fibroglandular microenvironment characterized by extracellular matrix remodeling and oxidative stress from the earliest stages of disease [35,41,42]. These conditions may promote sustained activation of Trx1-mediated redox responses throughout disease progression [43,44], providing a plausible explanation for the sustained elevation of circulating Trx1 across disease stages. This biological framework is consistent with the high sensitivity observed even in Stage 0 disease (Figure 2D) and the preferential elevation of Trx1 compared with other malignancies (Supplementary Figure S2). Exploratory SHAP (SHapley Additive exPlanations) analysis further supported the biological relevance of circulating Trx1, identifying Trx1 as the most influential feature within the XGBoost model, exceeding the contribution of BI-RADS classification and age. Although exploratory, these findings aligned with the observed diagnostic performance and support the biological relevance of the predefined Trx1 threshold [45].

Dense breast tissue remains one of the most persistent challenges in BC detection because it simultaneously increases BC risk and reduces mammographic sensitivity through masking effects [6,7,8,9]. Consistent with this well-recognized limitation, 77 of 308 BC (25.0%) in our cohort were initially assigned BI-RADS 0–3 categories (Figure 3A), and these classifications were more frequently observed among women with dense breasts (Figure 3B and Supplementary Table S3). Mammographic sensitivity declined with increasing breast density, whereas Trx1 maintained high sensitivity across breast-density categories (Figure 3C). This divergence likely reflects the fundamentally different information captured by imaging and circulating biomarkers. Mammography evaluates structural abnormalities within the breast, whereas circulating Trx1 reflects biological processes associated with tumor development. As a result, Trx1 appears largely independent of tissue density and may provide additional biological information in situations where imaging performance is reduced. The improved diagnostic performance associated with the combined mammography–Trx1 approach supports the broader concept that integrating imaging with biologically distinct biomarkers may improve cancer detection when imaging performance is limited [46,47,48]. Importantly, this complementary relationship was observed in the present study using a predefined Trx1 threshold across a large multicenter cohort, supporting the potential clinical applicability of Trx1 as an adjunctive biomarker in women with dense breasts. The clinical implications of these findings were further explored using decision curve analysis.

The DCA findings were broadly consistent with the diagnostic performance results. While improvements in AUC provide evidence of incremental predictive information, clinical integration ultimately depends on how additional variables influence decision thresholds and net clinical benefit [28]. Differences between strategies were modest when the full imaging cohort was considered, reflecting the strong discriminatory capacity of mammographic assessment in patients with highly suspicious imaging findings (Figure 4A). In contrast, greater separation between strategies was observed within the BI-RADS 0–3 subgroup, where diagnostic uncertainty is inherently higher (Figure 4B). In this setting, incorporation of Trx1 increased net benefit across a range of clinically relevant threshold probabilities, supporting the concept that biologically independent biomarkers may provide additional value when imaging findings are incomplete, negative, or only mildly suspicious [27,28]. Although DCA represents a model-based estimate rather than prospective clinical outcomes, the findings support the potential clinical utility of Trx1 as an adjunct to mammographic assessment, particularly in diagnostically challenging scenarios.

To further assess the independent diagnostic contribution of circulating Trx1, multivariable logistic regression analysis was performed (Supplementary Table S7). Among the variables included in the model, BI-RADS category showed the strongest association with BC, followed by circulating Trx1, whereas age was not independently associated with BC after adjustment. The estimated odds ratio for BI-RADS category should be interpreted with caution. Because women with BI-RADS 4 or 5 findings underwent additional diagnostic evaluation and, if malignancy was confirmed, were included in the BC cohort, all control participants were classified as BI-RADS 0–3. Consequently, BI-RADS exhibited exceptionally strong discriminatory power within this retrospective diagnostic cohort, resulting in a high estimated odds ratio and a tendency toward quasi-complete separation during model fitting. Nevertheless, circulating Trx1 remained independently associated with BC after adjustment for age and BI-RADS category, further supporting its independent diagnostic contribution beyond conventional imaging assessment.

The excellent diagnostic performance observed in the present study should be interpreted in the context of the study design. The retrospective case–control design and en-rollment of women undergoing diagnostic evaluation at tertiary referral centers may have contributed to higher estimates of diagnostic performance than would be expected in broader clinical practice. In particular, the study population consisted of women already referred for further evaluation because of suspected breast disease or abnormal clinical findings, rather than asymptomatic individuals undergoing routine screening (Figure 1). Consequently, both the prevalence of BC and the diagnostic separation between cases and controls were likely greater than would be encountered in population-based screening settings. Within this clinical context, however, the objective of the present study was not to evaluate Trx1 as a population-based screening biomarker. Instead, Trx1 was investigated as a biologically independent adjunct to mammographic assessment for patients already undergoing diagnostic assessment, where imaging findings may remain indeterminate despite clinical suspicion. Accordingly, the present findings are best interpreted as supporting the potential role of circulating Trx1 in supporting existing diagnostic pathways, rather than defining its performance in population-based screening. Despite these limitations, the principal objective of the present study was to determine whether circulating Trx1 provides biologically independent information beyond mammographic assessment within clinically relevant diagnostic pathways. Notably, Trx1 maintained high diagnostic performance across breast-density categories, disease stages, and molecular subtypes while providing incremental clinical utility in decision curve analysis. Therefore, although the diagnostic performance reported here may not directly translate to population-based screening settings, the findings support the potential role of circulating Trx1 as an adjunct to mammographic assessment, particularly in patients with persistent clinical suspicion despite indeterminate imaging findings.

Several limitations should be considered when interpreting these findings. First, this was a retrospective multicenter study rather than a prospective population-based screening study. As a result, the study population may not fully represent the disease prevalence and clinical heterogeneity encountered in routine screening practice. Accordingly, the diagnostic performance observed in the present study should be interpreted in the context of the study design and requires prospective validation. Furthermore, several host-related confounding factors that may influence oxidative stress biology were not uniformly available in this retrospective cohort and therefore could not be evaluated. Second, breast-density information was available for only a subset of participants, limiting the density-stratified analyses. Third, because the study population was derived from Korean centers, where dense breast tissue is relatively common, the generalizability of these findings to populations with different breast-density distributions remains to be established [49]. Future studies in diverse populations will be required to confirm the clinical utility and generalizability of Trx1 as an adjunct to mammographic assessment. Finally, Trx1 elevations were not restricted to BC and were also observed in several other malignancies. Although Trx1 cannot be regarded as a BC-specific biomarker, this does not preclude its clinical utility as an adjunct to mammographic assessment. In the present study, its diagnostic value was most apparent when interpreted in conjunction with imaging findings. Although the predefined Trx1 threshold showed strong diagnostic performance in this multicenter cohort, validation in independent prospective populations will be necessary to establish its broader clinical applicability.

## 5. Conclusions

Dense breast tissue remains a major challenge in breast cancer detection because it limits the sensitivity of imaging-based assessment while simultaneously increasing cancer risk. In this multicenter clinical validation study, circulating Trx1 showed robust diagnostic performance across mammographic categories, breast-density groups, tumor size, and molecular subtypes, supporting its role as a biologically independent biomarker. Integration of Trx1 with mammographic assessment improved diagnostic discrimination and provided complementary information beyond imaging alone. Rather than replacing established imaging modalities, Trx1 may serve as an independent source of biological information that helps refine breast cancer assessment, particularly in women with dense breasts and across early-stage disease. In clinical practice, this potential role may be most relevant for patients already undergoing diagnostic evaluation because of suspected breast disease but whose mammographic findings remain indeterminate (e.g., BI-RADS 0–3). In such situations, circulating Trx1 may provide complementary biological information to support decisions regarding further diagnostic evaluation within established diagnostic pathways.

## Figures and Tables

**Table 1:** Circulating Trx1 levels according to age and imaging findings.

Variables			N (%)	Trx1 (U/mL)	95% CI	*p* Value
Age	Normal and benign controls	<40	2 (0.60)	8.10 ± 6.01	−16.67	0.633
		40–49	57 (16.96)	6.39 ± 2.80	5.65–7.13	
		50–59	104 (30.95)	6.48 ± 3.02	5.89–7.07	
		60–69	126 (37.50)	6.63 ± 2.48	6.19–7.06	
		≥70	47 (13.99)	7.26 ± 3.51	6.23–8.29	
	Breast cancer patients	<40	27 (8.77)	29.41 ± 9.22	25.76–33.05	0.265
		40–49	101 (32.79)	29.79 ± 10.02	27.82–31.77	
		50–59	83 (26.95)	32.03 ± 9.45	29.96–34.11	
		60–69	61 (19.81)	28.37 ± 10.05	25.80–30.95	
		≥70	36 (11.69)	28.24 ± 11.47	24.36–32.12	
Mammography (BI-RADS)	Normal and benign controls	0	8 (2.38)	5.70 ± 2.97	3.22–8.18	0.364
		1	203 (60.42)	6.52 ± 2.88	6.12–6.92	
		2	118 (35.12)	6.81 ± 2.89	6.29–7.34	
		3	7 (2.08)	7.41 ± 2.43	5.16–9.66	
	Breast cancer patients	0	7 (2.27)	33.83 ± 11.46	23.23–44.43	0.155
		1	13 (4.22)	30.11 ± 6.17	26.38–33.83	
		2	34 (11.04)	30.56 ± 10.99	26.72–34.39	
		3	23 (7.47)	34.29 ± 12.13	29.04–39.53	
		4	112 (36.36)	28.93 ± 9.53	27.14–30.71	
		5	72 (23.38)	29.66 ± 10.63	27.16–32.16	
		6	47 (15.26)	29.33 ± 8.94	26.70–31.96	
Breast density	Breast cancer patients	A	10 (3.25)	33.83 ± 13.36	24.27–43.39	0.355
		B	65 (21.10)	28.11 ± 10.08	25.61–30.61	
		C	166 (53.90)	30.34 ± 9.82	28.84–31.85	
		D	20 (6.49)	31.34 ± 12.15	25.65–37.02	

**Table 2:** Clinical and molecular characteristics of breast cancer cases and circulating Trx1 levels.

Variables		N (%)	Trx1 (U/mL)	95% CI	*p* Value
Pathology	DCIS	39 (12.66)	28.85 ± 11.21	25.22–32.48	0.095
	IDC	230 (74.68)	30.09 ± 9.80	28.82–31.36	
	ILC	10 (3.25)	33.74 ± 9.51	26.94–40.54	
	Medulary	9 (2.92)	31.84 ± 9.29	24.71–38.98	
	Mucinous	7 (2.27)	20.21 ± 9.61	11.32–29.10	
	Papillary	6 (1.95)	35.27 ± 9.43	25.37–45.17	
	Tubular	3 (0.97)	33.66 ± 2.44	27.61–39.71	
	Metaplastic	1 (0.32)	30.57 ± 0.00	-	
	Microinvasive	2 (0.65)	20.69 ± 9.89	14.56–26.82	
	NOS	1 (0.32)	13.39 ± 0.00	-	
Biopsy (Grade)	1	82 (26.62)	29.64 ± 10.18	27.40–31.88	0.800
	2	119 (38.64)	30.05 ± 9.92	28.25–31.85	
	3	107 (34.74)	29.94 ±10.15	27.99–31.88	
Stage	0	39 (12.66)	28.85 ±11.21	25.22–32.48	0.337
	I	136 (44.16)	29.81 ± 9.60	28.18–31.44	
	II	108 (35.06)	31.09 ± 10.20	29.15–33.04	
	III	25 (8.12)	26.87 ± 9.49	22.95–30.78	
	IV	0 (0.00)	-	-	
T Stage	Tis	39 (12.66)	28.85 ± 11.21	25.22–32.48	0.871
	T1	159 (51.62)	29.82 ± 9.57	28.32–31.32	
	T2	104 (33.77)	30.59 ± 10.10	28.63–32.56	
	T3	6 (1.95)	26.86 ± 14.45	11.70–42.02	
N Stage	N0	228 (74.03)	29.82 ± 9.77	28.54–31.09	0.374
	N1	55 (17.86)	31.62 ± 11.14	28.61–34.63	
	N2	14 (4.55)	27.27 ± 9.26	21.92–32.62	
	N3	11 (3.57)	26.35 ± 10.20	19.50–33.21	
M Stage	M0	308 (100.00)	29.90 ± 10.04	28.78–31.03	-
Tumor size	<5 mm	14 (5.36)	29.12 ± 6.83	25.54–32.70	0.972
	5–10 mm	69 (26.44)	30.48 ± 11.73	27.71–33.25	
	10–20 mm	110 (42.15)	30.02 ± 9.55	28.23–31.80	
	>20 mm	62 (23.75)	29.47 ± 9.79	27.03–31.91	
	No lesion identified	6 (2.30)	33.27 ± 14.67	21.53–45.01	
Molecular Subtype	Luminal A	21 (6.82)	27.97 ± 9.99	23.42–32.52	0.907
	Luminal B	218 (70.78)	30.00 ± 10.03	28.66–31.34	
	HER-2/neu	55 (17.86)	30.29 ± 10.36	27.49–33.09	
	TNBC	14 (4.55)	30.11 ± 9.96	24.36–35.87	
Ki67	<15%	163 (52.92)	28.84 ± 9.98	27.29–30.38	0.079
	≥15%	145 (47.08)	31.10 ± 10.00	29.46–32.74	
p53	Positive	243 (78.90)	29.74 ± 10.24	28.45–31.03	0.256
	Negative	41 (13.31)	29.09 ± 9.71	26.03–32.16	
	N/A	24 (7.79)	32.90 ± 8.24	29.42–36.38	

Abbreviations: DCIS; ductal carcinoma in situ, IDC; invasive ductal carcinoma, ILC; invasive lobular carcinoma, NOS; no special type, Tis; T category staging of breast cancer for DCIS or Paget’s diseases of the breast with no related tumor, HER-2/neu; human epidermal growth factor receptor-2, TNBC; triple-negative breast cancer.

## Data Availability

The datasets generated and/or analyzed during the current study are available from the corresponding author on reasonable request. Access to individual-level data may be restricted due to ethical and privacy considerations.

## Supplementary Materials

The following supporting information can be downloaded at: https://www.mdpi.com/article/10.3390/cancers18152416/s1, Figure S1: Participant allocation and imaging-based clinical assessment framework. Figure S2: Circulating Trx1 concentrations across breast cancer, other malignancies, inflammatory diseases, benign breast disease, and healthy controls. Figure S3: SHAP analysis of the combined mammography–Trx1 model in the imaging comparison cohort (*n* = 597).; Table S1: Baseline demographic characteristics of the clinically enrolled study population (*n* = 1032), Table S2: Quantitative characteristics of circulating Trx1 in the primary diagnostic performance cohort (Cohort I), Table S3: Distribution of breast density across BI-RADS categories in the density-stratified performance cohort (Cohort II), Table S4: Comparative diagnostic performance according to breast density in the density-stratified performance cohort (Cohort II), Table S5: Comparative contribution of mammography and Trx1 in the imaging comparison cohort (Cohort III), Table S6: Circulating Trx1 concentrations across malignant and non-malignant conditions in the cross-disease specificity cohort (Cohort IV).

## Abbreviations

The following abbreviations are used in this manuscript:

BC	Breast Cancer
Trx1	Thioredoxin 1
ROC	Receiver Operating Characteristic
AUC	Area Under the Curve
ANOVA	Analysis of Variance
TNM	Tumor, Node, Metastasis
CA 15-3	Cancer Antigen 15-3
CEA	Carcinoembryonic Antigen
ROS	Reactive Oxygen Species
BI-RADS	Breast Imaging Reporting and Data System
DCA	Decision Curve Analysis
SHAP	SHapley Additive exPlanations
AUPRC	Area Under the Precision-Recall Curve
PPV	Positive Predictive Value
NPV	Negative Predictive Value
DCI	Ductal Carcinoma In Situ
IDC	Invasive Ductal Carcinoma
ILC	Invasive Lobular Carcinoma
TNBC	Triple-Negative Breast Cancer
COPD	Chronic Obstructive Pulmonary Disease
NBC	Non-Breast Cancer
ECM	Extracellular Matrix
ELISA	Enzyme-Linked Immunosorbent Assay
