# Supplementary material for: Circulating Thioredoxin 1 as an Adjunct to Mammography for Breast Cancer Detection: A Multicenter Clinical Validation Study
Source: Cancers (Basel). 2026 Jul 27;18(15):2416. doi: 10.3390/cancers18152416 (PMC13465374; doi:10.3390/cancers18152416)
Supplement: Supplementary file 1 [file cancers-18-02416-s001.zip › Supplementary Tables.pdf]

Supplemental Table S1. Baseline demographic characteristics of the clinically enrolled study population (n=1,032)

Variables	BC	Benign+NBC	Other cancer
**Age**			
N (%)	308 (29.84)	336 (32.56)	388 (37.60)
Mean ± SD	54.00 ± 11.21	58.69 ± 9.13	62.1 ± 12.1
Median	52	60	64
Min/Max	25/80	39/78	23/80
**Sex (N, %)**			
Male	0 (0.00)	0 (0.00)	228 (58.76)
Female	308 (100.00)	336 (100.00)	160 (41.24)

Abbreviations: BC; breast cancer, NBC; non-breast cancer

Supplemental Table S2. Quantitative characteristics of circulating Trx1 in the primary diagnostic performance cohort (Cohort I)

		Breast cancer		Total
		**Malignancy**	**Non-malignancy**	
**Trx1 Results**	**Elevated**	297	9	306
	**Not elevated**	11	327	338
	**Total**	308	336	644

Clinical Performance Parameters	N=644	95% Confidence interval
Clinical Sensitivity	96.43% (297/308)	93.7-98.2%
Clinical Specificity	97.32% (327/336)	95.0-98.8%
Negative Predictive Value (NPV)	96.75% (327/338)	94.3-98.2%
Positive Predictive Value (PPV)	97.06% (297/308)	94.5-98.4%
Area Under Curve (AUC)	0.985	0.974-0.996

Supplemental Table S3. Distribution of breast density across BI-RADS categories in the density-stratified performance cohort (Cohort II)

BI-RADS	Density A	Density B	Density C	Density D	Total
**0**	0 (0%)	3 (4.62%)	4 (2.41%)	0 (0%)	7
**1**	0 (0%)	1 (1.54%)	11 (6.63%)	1 (5%)	13
**2**	1 (10%)	3 (4.62%)	24 (14.46%)	6 (30%)	34
**3**	4 (40%)	4 (6.15%)	14 (8.43%)	1 (5%)	23
**4**	3 (30%)	30 (46.15%)	69 (41.57%)	10 (50%)	112
**5**	2(20%)	24 (36.92%)	44 (26.51%)	2 (10%)	72
**Total**	10	65	166	20	261

Supplemental Table S4. Comparative diagnostic performance according to breast density in the density-stratified performance cohort (Cohort II)

Density Grade	N	Trx1 (U/mL)	Sensitivity (%)				
			CA15-3	CEA	Trx1	Mammo	Mammo+Trx1
**A (Fatty)**	10	33.83±13.36	0.00	30.00	90.00	50.00	90.00
**B (Scattered)**	63	28.50±9.95	4.76	3.17	92.06	85.71	100.00
**C (Heterogeneously dense)**	165	30.34±9.85	0.61	6.06	99.39	67.88	99.39
**D (Extremely dense)**	20	31.34±12.15	5.00	0.00	95.00	60	100.00
**A, B**	73	29.23±10.53	4.11	6.85	91.78	80.82	98.63
**C, D**	185	30.44±10.09	1.08	5.41	98.92	67.03	99.46
**Total**	258	29.23±10.53	1.94	5.81	96.90	70.93	99.23

Abbreviations: Mammo; mammography

Supplemental Table S5. Comparative contribution of mammography and Trx1 in the imaging comparison cohort (Cohort III)

	**Mammography**	**Trx1 Test**	**Combined Test (Mammography+Trx1 test)**
Sample size	582	582	582
Positive group	254 (43.64%)	254 (43.64%)	254 (43.64%)
Negative group	328 (56.36%)	328 (56.36)	328 (56.36%)
Area under the ROC curve (AUC)	0.862	0.969	0.980
Standard error	0.014	0.00736	0.00565
95% Confidence interval	0.832-0.889	0.951-0.981	0.966-0.990
95% Bootstrap CI	0.500-0.500	0.500-0.500	0.500-0.500
Z statistic	25.788	63.661	85.005
Significance level P (Area=0.5)	<0.0001	<0.0001	<0.0001
Youden index J	0.7244	0.9371	0.9607
95% Confidence interval	0.6693-0.7717	0.9044-0.9633	0.9337-0.9799
Associated criterion	>0	>0	>0
95% Confidence interval	>0->0	>0->0	>0->0
Sensitivity	72.44	96.46	98.82
Specificity	100.00	97.26	97.26

Supplemental Table S6. Circulating Trx1 concentrations across malignant and non-malignant conditions in the cross-disease specificity cohort (Cohort IV)

Diseases	N (%)	Trx1(U/ml)	95% CI	Fold-change	P-value
Breast cancer	437 (22.99)	32.28±10.58	31.29 - 33.27	-	<0.001
Women without breast cancer	426 (22.41)	6.90±4.48	6.47 - 7.33	0.21	
Benign breast disease	82 (4.31)	7.06±3.00	6.40 - 7.00	0.22	
Ovarian cancer	150 (7.89)	12.67±11.63	10.88 - 14.55	0.39	
Cervical cancer	100 (5.26)	14.28±10.18	12.26 - 16.30	0.44	
Lung cancer	178 (9.36)	15.00±12.03	13.22 - 16.78	0.46	
Stomach cancer	188 (9.89)	22.31±10.74	20.77 - 23.86	0.69	
Colon cancer	200 (10.52)	21.44±10.03	20.04 - 22.84	0.66	
Pancreatic cancer	37 (1.95)	18.84±16.65	13.29 - 24.39	0.58	
Kidney cancer	10 (0.53)	21.68±17.46	9.19 - 34.16	0.67	<0.01
Asthma	18 (0.95)	15.09±12.51	8.87 - 21.32	0.47	<0.001
Rheumatoid arthritis	20 (1.05)	10.52±6.04	7.70 - 13.35	0.33	
COPD	15 (0.79)	10.51±6.01	7.18 - 13.83	0.33	
Psoriasis	15 (0.79)	15.59±12.51	8.67 - 22.52	0.48	
Inflammatory disease, suspected	17 (0.89)	14.13±9.90	9.04 - 19.22	0.44	
Sjogren's syndrome	8 (0.42)	11.7±10.05	3.30 - 20.10	0.36	

Supplemental Table S7. Multivariable logistic regression analysis for breast cancer diagnosis

Variable	Adjusted OR	95% CI	P-value
Age (per year)	0.924	0.852-1.001	0.054
BI-RADS (per category increase)	14.02	4.48-43.85	<0.001
Trx1 (per 1 U/mL increase)	1.76	1.45-2.13	<0.001
