# Supplementary material for: Circulating Thioredoxin 1 as an Adjunct to Mammography for Breast Cancer Detection: A Multicenter Clinical Validation Study
Source: Cancers (Basel). 2026 Jul 27;18(15):2416. doi: 10.3390/cancers18152416 (PMC13465374; doi:10.3390/cancers18152416)
Supplement: Supplementary file 1 [file cancers-18-02416-s001.zip › Supplementary Figures.pdf]

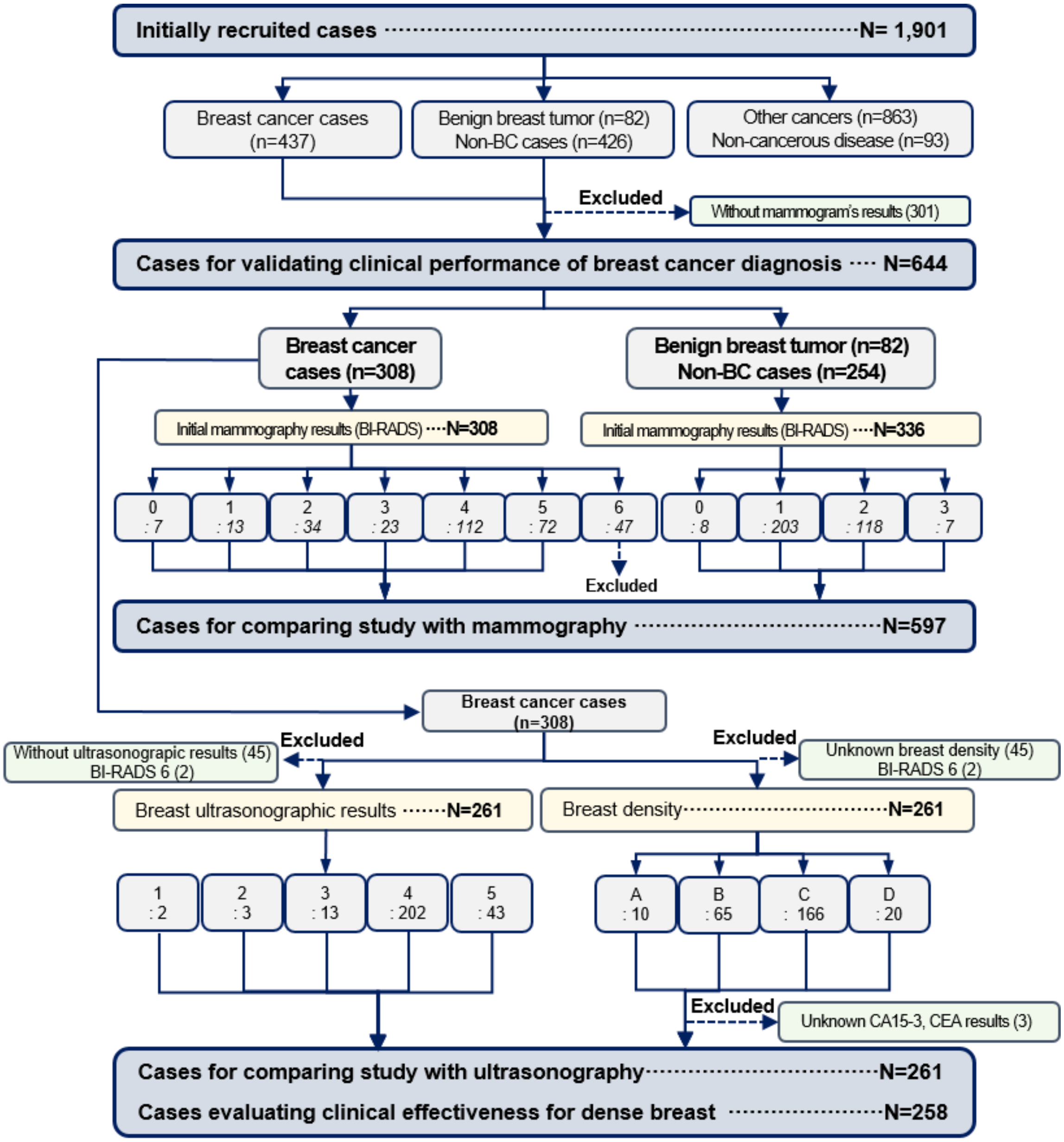

Supplemental Figure S1. Participant allocation and imaging-based clinical assessment framework. Flow diagram showing participant selection and derivation of the imaging-based analytical cohorts. Among 1,901 recruited participants, 644 individuals with available mammographic data were included for evaluation of breast cancer diagnostic performance. Subsequent exclusions based on imaging availability, breast density information, and biomarker measurements yielded the cohorts used for mammography- and density-stratified analyses.

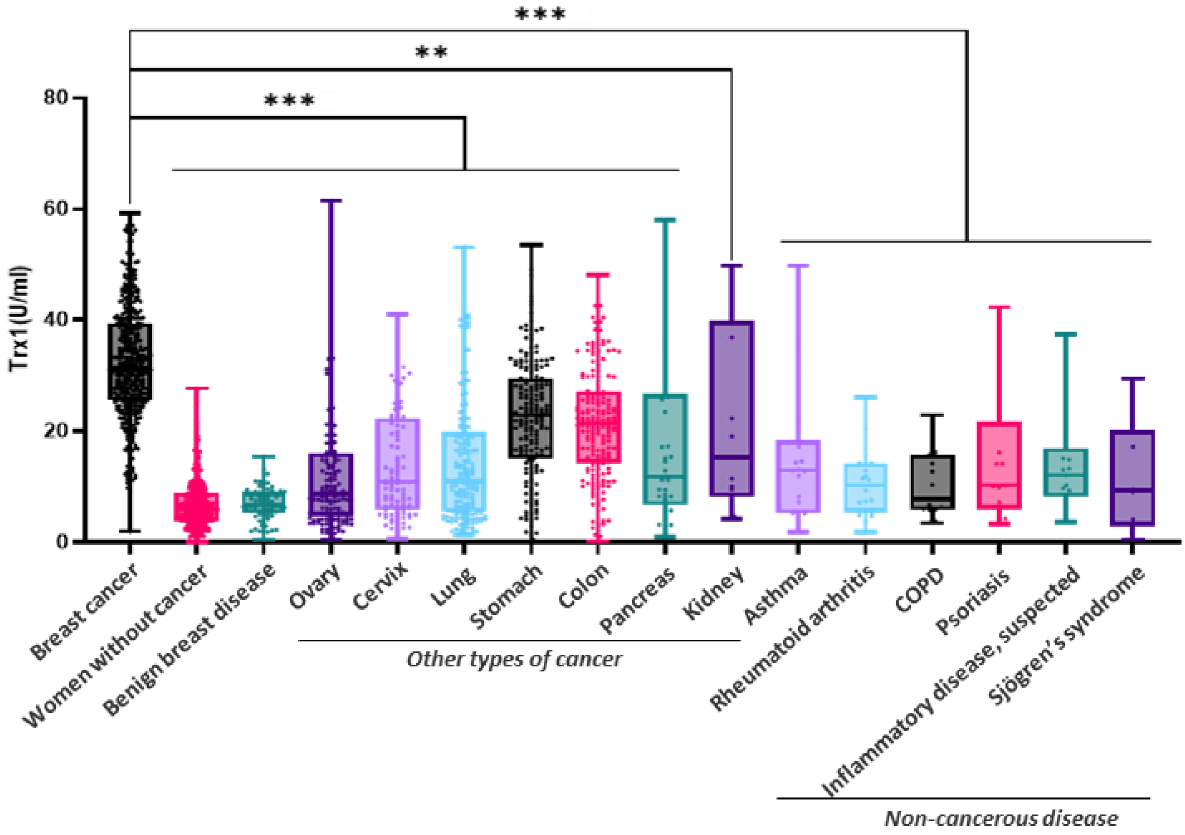

Supplemental Figure S2. Circulating Trx1 concentrations across breast cancer, other malignancies, inflammatory diseases, benign breast disease, and healthy controls. Box-and-whisker plots show the distribution of circulating Trx1 concentrations in each group. The central line indicates the median, and boxes represent the interquartile range. **P<0.01; ***P<0.001. COPD, chronic obstructive pulmonary disease.

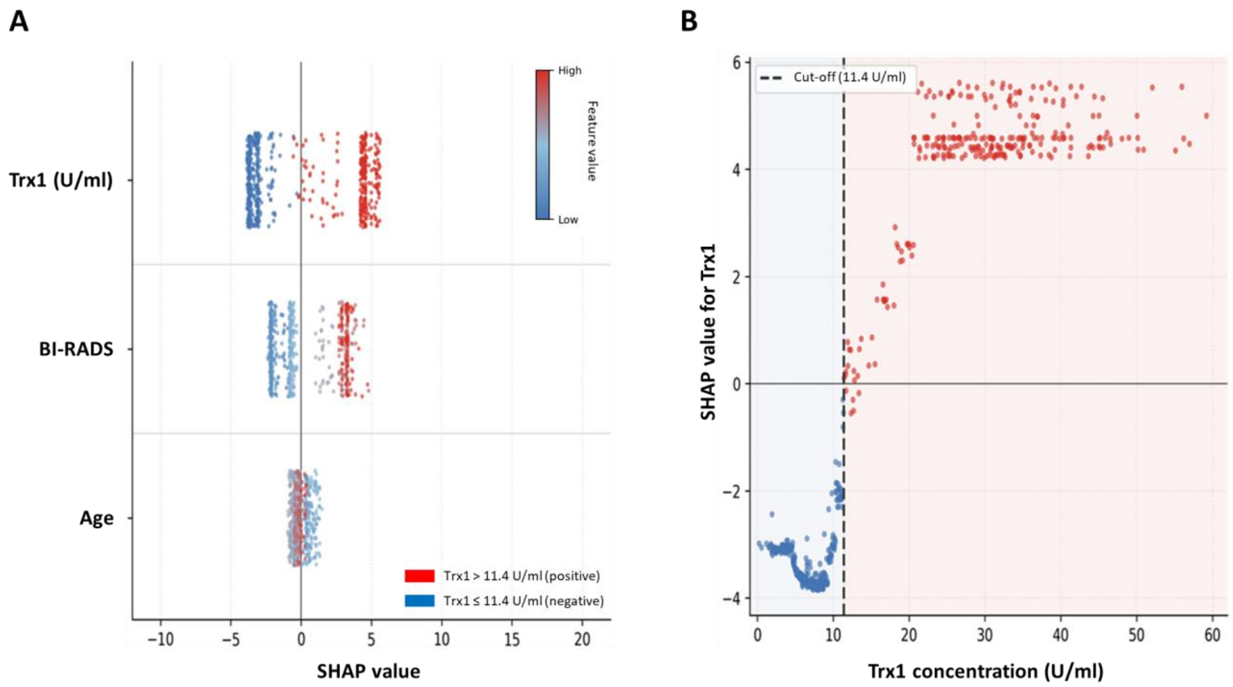

Supplemental Figure S3. SHAP analysis of the combined mammography–Trx1 model in the imaging comparison cohort (n=597). (A) SHAP summary plot showing the relative contribution of Trx1, BI-RADS category, and age to model predictions. Colors indicate feature values (red, high; blue, low). (B) SHAP dependence plot demonstrating the relationship between circulating Trx1 concentration and its contribution to model output. The dashed vertical line indicates the predefined Trx1 cut-off (11.4 U/ml).
